# Green, Efficient Detection and Removal of Hg^2+^ by Water-Soluble Fluorescent Pillar[5]arene Supramolecular Self-Assembly

**DOI:** 10.3390/bios12080571

**Published:** 2022-07-27

**Authors:** Xiaomei Jiang, Lingyun Wang, Xueguang Ran, Hao Tang, Derong Cao

**Affiliations:** 1 Key Laboratory of Functional Molecular Engineering of Guangdong Province, School of Chemistry and Chemical Engineering, South China University of Technology, 381 Wushan Road, Guangzhou 510641, China; 201910104286@mail.scut.edu.cn (X.J.); haotang@scut.edu.cn (H.T.); drcao@scut.edu.cn (D.C.); 2 Institute of Animal Science, Guangdong Academy of Agricultural Sciences, Ministry of Agriculture Key Laboratory of Animal Nutrition and Feed Science in South China, State Key Laboratory of Livestock and Poultry Breeding, Guangzhou 510641, China; rxg59@aliyun.com

**Keywords:** supramolecular self-assembly, fluorescence sensing, water-soluble pillar[5]arene, mercury(II) detection, diketopyrrolopyrrole

## Abstract

Developing a water-soluble supramolecular system for the detection and removal of Hg^2+^ is extremely needed but remains challenging. Herein, we reported the facile construction of a fluorescent supramolecular system (**H⊃G**) in 100% water through the self-assembly of carboxylatopillar[5]arene sodium salts (**H**) and diketopyrrolopyrrole-bridged bis(quaternary ammonium) guest (**G**) by host–guest interaction. With the addition of Hg^2+^, the fluorescence of **H⊃G** could be efficiently quenched. Since Hg^2+^ showed synergistic interactions (coordination and Hg^2+^- cavity interactions with **G** and **H**, respectively), crosslinked networks of **H⊃G**@Hg^2+^ were formed. A sensitive response to Hg^2+^ with excellent selectivity and a low limit of detection (LOD) of 7.17 × 10^−7^ M was obtained. Significantly, the quenching fluorescence of **H⊃G**@Hg^2+^ can be recovered after a simple treatment with Na_2_S. The reusability of **H⊃G** for the detection of Hg^2+^ ions was retained for four cycles, indicating the **H⊃G** could be efficiently used in a reversible manner. In addition, the **H⊃G** could efficiently detect Hg^2+^ concentration in real samples (tap water and lake water). The developed supramolecular system in 100% water provides great potential in the treatment of Hg^2+^ detection and removal for environmental sustainability.

## 1. Introduction

Mercury ion (Hg^2+^), as one of the most toxic heavy metal ions in the environment and aquatically derived food, has become an important worldwide pollution problem. Hg^2+^ is a neurotoxin and can cause severe adverse effects on human health [1,2,3]. More importantly, as the most stable and toxic form of mercury, methylmercury in aquatic food chains results in serious damage to the heart, brain, kidneys and immune systems. The maximum contaminant level of Hg^2+^ (10 µg/L) in wastewater discharge was established by World Health Organization (WHO) [4]. Thus far, several functional materials, such as hydrogels, nanoparticles, covalent organic frameworks, porous aromatic frameworks, metal–organic frameworks and others, have shown potential for the possible detection and removal of Hg^2+^ from wastewater [5,6,7,8,9,10].

Among the various methods for Hg^2+^ recognition, fluorescent self-assembled materials with advanced architectures and stimuli-responsive properties by the supramolecular host–guest interactions have attracted considerable interest [11,12]. They can sensitively and selectively detect targeted analytes with excellent simplicity and good efficiency [13,14]. Pillar[n]arenes are especially popular supramolecular host molecules for their ability to selectively bind different kinds of guests [15,16]. They have a pillar-like framework and multiple self-assembly driving forces such as hydrophobic/hydrophilic, π⋯π, C-H⋯π, cation⋯π interactions, etc. Thus far, the pillararene-based fluorescent polymer, supramolecular organic framework, gel and supramolecular assembly as Hg^2+^ sensors have been studied by several groups [17,18,19,20,21,22,23,24]. For instance, Yuan’s group reported pillar[5]arenes bearing phosphine oxide pendents as Hg^2+^ selective receptors [17]. Wu’s and Yang’s group developed pillar[5]arene and pillar[6]arene-based aggregation-induced emission-active supramolecular system for detection and removal of Hg^2+^, respectively [18,21]. Lin’s group investigated pillar[5]arene-based polymer and gel as Hg^2+^ fluorescent sensors [22]. Although considerable research efforts for the development of Hg^2+^ supramolecular chemosensors have been made, the participation of organic solvents in these cases is needed, which is unfavorable for Hg^2+^ detection in biological and environmental systems. Moreover, the complicated and tedious synthesis of functional pillararenes is involved. For example, the introduction of Hg^2+^ recognition sites such as thymine, N or S moiety in pillararene is time-costing and cumbersome. More importantly, strong interference from other metal ions is encountered due to the noncovalent interactions sensing mechanism. Therefore, developing water-soluble pillararene-based supramolecular fluorescent materials with excellent Hg^2+^ detection performance in pure water is extremely needed but remains challenging. To the best of our knowledge, a fluorescent supramolecular system in pure water for Hg^2+^ detection and removal has not yet been reported.

Herein, we constructed a supramolecular fluorescent system in pure water, which aims to accomplish the goal of Hg^2+^ sensing and removal in one pot. Some design intentions are listed as follows. (1) Water-soluble carboxylatopillar[5]arene possessed five carboxylate groups on each rim and good binding ability toward guest molecules [25,26,27,28,29,30,31,32,33,34,35,36], which was selected as a supramolecular host. It may also have cation⋯π and electrostatic interactions with Hg^2+^. (2) Given that Hg^2+^ possesses a high affinity for N and S, cationic thienyl functionalized diketopyrrolopyrrole (DPP) guest with two six-carbon alkyl chains was selected. It would form a fluorescent supramolecular host–guest system with pillar[5]arene host and possess coordination ability with Hg^2+^. (3) Through synergistic interactions between Hg^2+^ and host/guest, excellent selectivity and sensitivity would be obtained by fluorescence signal change. By taking these factors into consideration and our research interest in supramolecular fluorescent chemosensors for pollutants [37,38,39,40], water-soluble fluorescent pillar[5]arene supramolecular self-assembly was constructed by carboxylatopillar[5]arene sodium salts (**H**) and cationic DPP quaternary ammonium (**G**). The as-prepared supramolecular host–guest system (**H⊃G**) showed yellow emission with a multi-layered nanostructure. The synergistic interactions between Hg^2+^ and **H⊃G** produce crosslinked networks. The intertwined supramolecular complex **H⊃G**@Hg^2+^ exhibited strong aggregation-caused quenching (ACQ) fluorescence. This enables convenient detection of Hg^2+^ and facile monitoring of the removal procedure. Significantly, the quenching fluorescence of **H⊃G**@Hg^2+^ can be recovered after treatment with Na_2_S. As reversible hybrid materials, **H⊃G** could be efficiently used in the treatment of Hg^2+^ detection and removal in a reusable manner.

## 2. Results and Discussion

### 2.1. Self-Assembly Behavior of G in Water

**Scheme 1:**
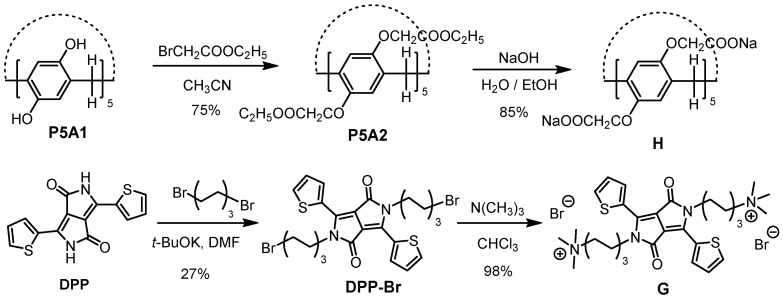
Synthesis of host molecule **H** and guest molecule **G**.

A simpler and modified synthetic method of **H** was developed (Scheme 1). In brief, etherification of OH functionalized pillar[5]arene (**P5A1**) afforded ethoxycarbonylmethoxy-substituted pillar[5]arene (**P5A2**). Then, the hydrolysis of **P5A2** with NaOH by a one-step reaction generated **H** in a high yield. However, a three-steps reaction (hydrolysis, acidification and neutralization) from **P5A2** to **H** was needed in the previous synthesis [36]. In addition, the synthesis of **G** was more straightforward than the reported method [41]. In our case, the N-alkylation and following substitution reaction afforded water-soluble **G**. On the contrary, tedious protection/deprotection reactions of the amino group and HPLC purification of **G** were performed [41]. The ^1^H and ^13^C NMR spectra of **H** and **G** are shown in Figures S1–S4.

**Figure 2:**
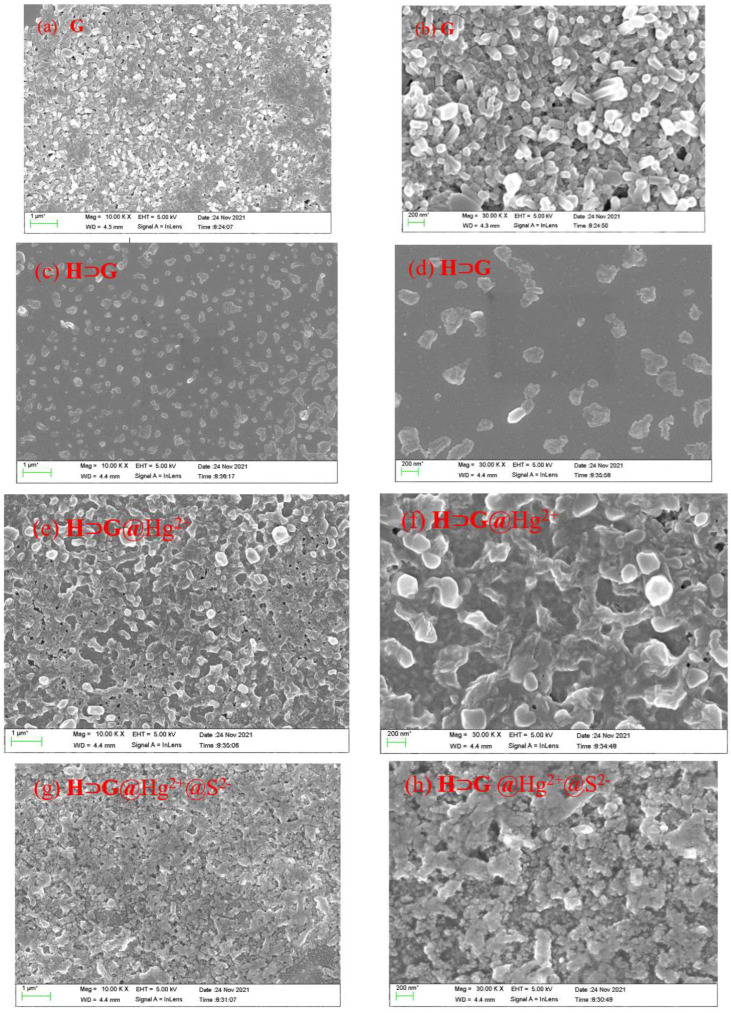
SEM and enlarged SEM images of the **G** (**a**,**b**), **H⊃G** (**c**,**d**), **H⊃G**@Hg^2+^ (**e**,**f**) and **H⊃G**@Hg^2+^@S^2−^ (**g**,**h**). [**H**] = 2 × 10^−5^ M, [**G**] = 10^−5^ M, [Hg^2+^] = 2 × 10^−5^ M, [S^2−^] = 4 × 10^−5^ M.

The aqueous solution of **G** exhibited a strong shoulder peak at 507/532 nm and a weak band at 350 nm (Figure S5). Its fluorescence showed a shoulder emission at 560 and 600 nm as well as a weak emission at 650 nm (Figure S5). Since **G** contained a hydrophobic DPP core and two hydrophilic, flexible tails, it tended to form nanoaggregates in water because of the strong π-π stacking among DPP units and the hydrophilic/hydrophobic interaction. ^1^H NMR spectrum of **G** proved this assumption, where the chemical shift of hydrogens on thienyl rings of **G** did not split well and showed broad peaks due to aggregation (Figure S3). The SEM images revealed that **G** in water formed tight packing with plenty of nanorods form (Figure 2a,b).

### 2.2. Host–Guest Complexation Studies in Water

**Figure 1:**
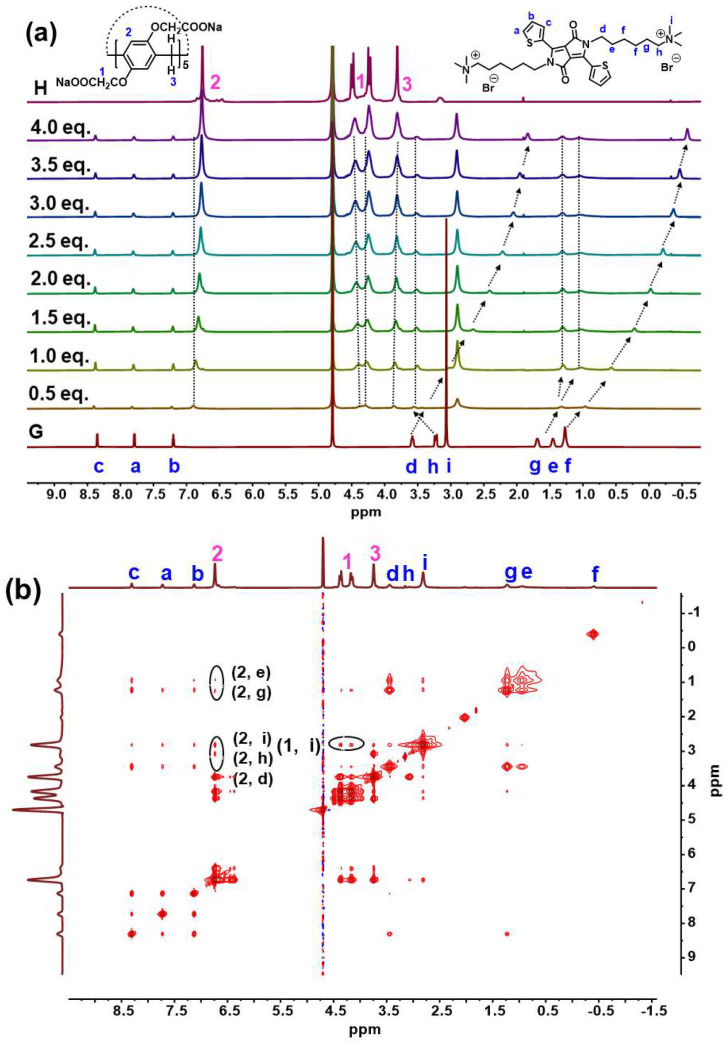
(**a**) ^1^H NMR titration spectra (500 MHz, D_2_O) of **G** (8.3 mM) with different equivalents of **H**. The deuterated water has a chemical shift of 4.79 ppm; (**b**) NOESY spectrum (500 MHz, D_2_O) of **H⊃G** ([**H**] = 33.2 mM, [**G**] = 8.3 mM).

The host–guest complexation was investigated between **G** and **H** in D_2_O by ^1^H NMR titration spectrum. Figure 1a shows the ^1^H NMR spectra of **G** in D_2_O recorded in the absence and in the presence of various amounts of **H**. Upon addition of **H**, alkyl protons signals of **G** displayed a clear upfield shift in sharp contrast with pure **G** (Δδ = −1.76, −0.33, −0.30, −0.37, −1.62 ppm for protons H_f_, H_e_, H_g_, H_h_, H_d_, respectively), indicating the shielding effect of pillar[5]arene cavity and the existence of inclusion complexation. With regard to the ^1^H NMR spectrum of **H**, obvious downfield shift (Δδ = 0.14 ppm for protons H_2_ in phenyl) further confirmed the host–guest inclusion complexation between **H** and **G**. Furthermore, 2D NOESY NMR experiments were performed to investigate the relative spatial positions of protons in this host–guest complex (Figure 1b). It exhibited unequivocal correlation peaks between the signals of H_d_-H_i_ on the alkyl chain of the **G** and H_2_ on the phenyl of **H** on viewing the NOESY cross-peaks.

SEM was conducted to characterize the morphology of **H⊃G**. As shown in Figure 2c,d, the scattered **H⊃G** assemblies with multi-layered nanostructures were observed, which were totally different from that of **G**. We speculated that the formation of the **H⊃G** complex was mainly driven by multiple electrostatic interactions, hydrophobic interactions and π-π stacking interactions in aqueous solution. The cooperativity of these noncovalent interactions made **G** be placed inside the cavity of **H** successfully.

**Figure 3:**
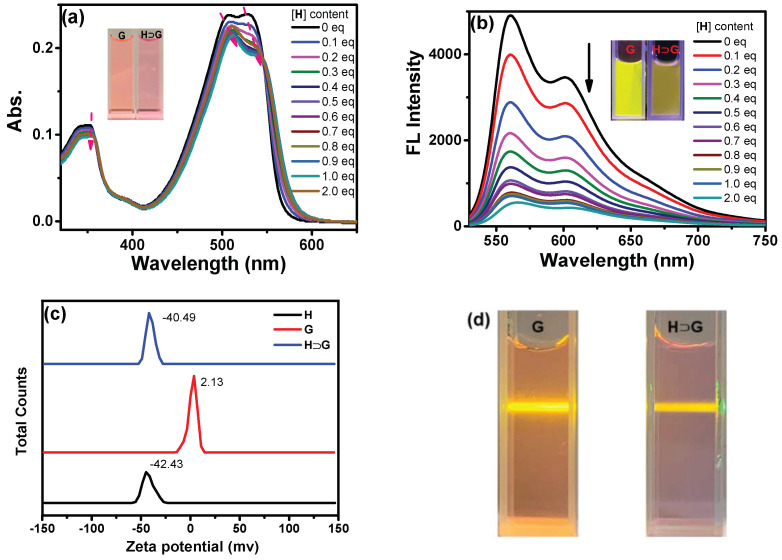
The (**a**) UV-vis and (**b**) emission spectra of **G** aqueous solution in the presence of increasing amounts of **H** aqueous solution ([**G**] = 10^−5^ M, λ_ex_ = 510 nm). Insert: Photos of **G** and **H⊃G** self-assembly under daylight and 365 nm irradiation; (**c**) The zeta potential of **H** (10^−5^ M), **G** (10^−5^ M) and **H⊃G** aqueous solution; (**d**) The photographs of Tyndall effect of aqueous solution of **G** and **H⊃G** ([**H**] = 2 × 10^−5^ M, [**G**] = 10^−5^ M).

The complexation between **H** and **G** also generated UV-vis and emission spectral changes. Upon addition of **H**, the redshifted bands from 507 to 512 and 532 to 538 nm with decreased absorption for **G** were shown, which was ascribed to complexation-induced charge transfer absorption (Figure 3a). Moreover, the absorbance at 350 nm decreased, and the spectrum became broader. As a result, a color change from light pink to pink could be seen, suggesting the formation of a new aggregation state of **H⊃G**. At the same time, the addition of **H** led to fluorescence quenching of **G** to some extent (Figure 3b). For example, the absolute fluorescence quantum yield of **G** decreased from 39.1% to 3.7% in the absence and presence of 2 equiv. **H**, respectively. The possible reason can be ascribed to the fact that multi-layered nanostructures gave closer DPP core stacking and resulted in fluorescence quenching of **G**. At the same time, the zeta potential of −42.43, 2.13 and −40.49 mV for **H, G** and **H⊃G** was found, respectively (Figure 3c). Moreover, an obvious Tyndall effect of **H⊃G** was shown (Figure 3d). All in all, a host–guest system (**H⊃G**) was constructed successfully by adding **H** into **G** aqueous solution, which could induce the change in the aggregation state and fluorescence behavior of **G**.

In theory, one **G** molecule can be complex with two **H** molecules. Assuming 1:2 inclusion complexation stoichiometry between **H** and **G**, the association constants (*K*_a_) could be calculated by using a non-linear least-squares curve-fitting method by use of fluorescence titrations of **G** with **H**. The plot of fluorescence change as a function of [**H**] gave an excellent fit, verifying the validity of the 1:2 inclusion complexation stoichiometry (Figure S6). The *K*_a_ value of **H⊃G** was determined to be 4.8 × 10^5^ M^−2^, indicating strong complexes were formed.

### 2.3. Detection of Hg^2+^ with H⊃G

**Figure 4:**
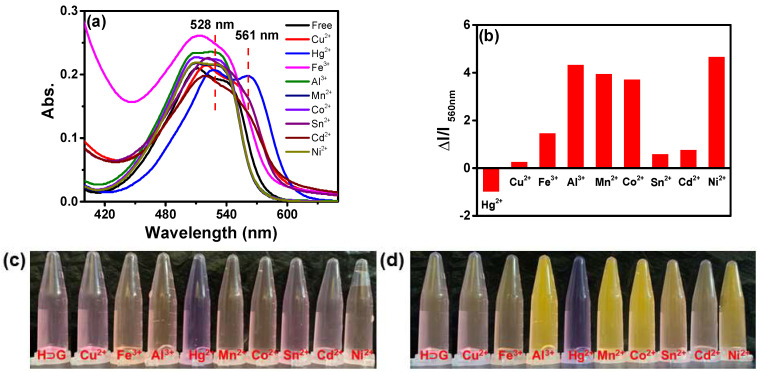
(**a**) UV-vis and (**b**) emission spectra of **H**⊃**G** self-assembly in aqueous solution with the addition of 50 equiv. of different metal ions ([**G**] = 10^−5^ M, [**H**] = 2 × 10^−5^ M, λ_ex_ = 510 nm). The photos of **H**⊃**G** in presence of various metal ions under (**c**) daylight and (**d**) 365 nm irradiation.

A series of metal ions including Hg^2+^, Cu^2+^, Fe^3+^, Al^3+^, Mn^2+^, Co^2+^, Sn^2+^, Cd^2+^, Ni^2+^, Au^+^, Ba^2+^, Ca^2+^, Ag^+^, K^+^, Fe^2+^, Mg^2+^, Pb^2+^, Na^+^, Zn^2+^ and Cr^3+^ were separately added to **H⊃G** in 100% water. As shown in Figure 4a, only Hg^2+^ gave a notable red-shift from 512 to 528 nm and 538 to 561 nm in UV-vis spectra. Meanwhile, the absorption spectrum became broader and extended to 650 nm. As a result, the solution was transformed from pink to purple, which can be observed by naked eyes (Figure 4c). According to a previous study [42], these changes indicated the formation of a typical charge–transfer complex between **H⊃G** and Hg^2+^. On the contrary, other metal ions induced absorption intensity change in **H⊃G** to some extent (Figure S7a), but its maximum absorption peaks at 512 and 538 nm showed no obvious red-shift or blue-shift.

In addition, only Hg^2+^ could induce the complete fluorescence quenching of **H⊃G**. The absolute fluorescence quantum yield of **H⊃G** decreased from 3.7% to 0.02% in the absence and presence of 10 equiv. Hg^2+^. The addition of other metal ions did not lead to fluorescence quenching of **H⊃G**. Contrarily, Cu^2+^, Sn^2+^, Fe^3+^, Cd^2+^ gave less than 2-fold fluorescence enhancement. Al^3+^, Mn^2+^, Co^2+^, Ni^2+^ could induce 4~5 fold fluorescence enhancement (Figure 4b,d). As shown in Figure S7b, Ca^2+^, Mg^2+^, Ba^2+^ and Pb^2+^ could induce fluorescence enhancement of **H**⊃**G** to a different extent. Fe^2+^, Ag^+^, Na^+^, Au^+^ and K^+^ failed to give obvious fluorescence changes in emission spectra. Moreover, the anti-interference experiment indicated the presence of other competing cations did not affect the response of **H⊃G** towards Hg^2+^ (Figure S8). Thus, based on UV-vis and emission change in **H⊃G** in the presence of Hg^2+^, the visual and highly selective detection of Hg^2+^ was obtained.

**Figure 5:**
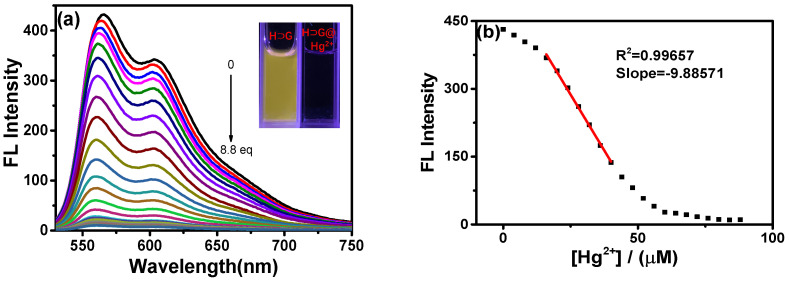
(**a**) Fluorescence spectra of **H⊃G** self-assembly in the presence of increasing amounts of Hg^2+^; (**b**) Plot of fluorescence intensity of **H⊃G** self-assembly at 565 nm against the concentration of Hg^2+^ (λ_ex_ = 510 nm).

In order to investigate the sensitivity of **H⊃G** for Hg^2+^, fluorescence titration experiments were monitored. The fluorescence emission intensity at 560 gradually decreased with an increase in Hg^2+^ content (Figure 5a). The limit of detection (LOD) of the **H⊃G** system for Hg^2+^ was determined to be 7.17 × 10^−7^ M according to the 3σ/K method (Figure 5b). The detection performance was similar to or superior to reported pillararenes-based supramolecular systems (Table 1).

### 2.4. Detection Mechanism of Hg^2+^ with H⊃G

**Figure 6:**
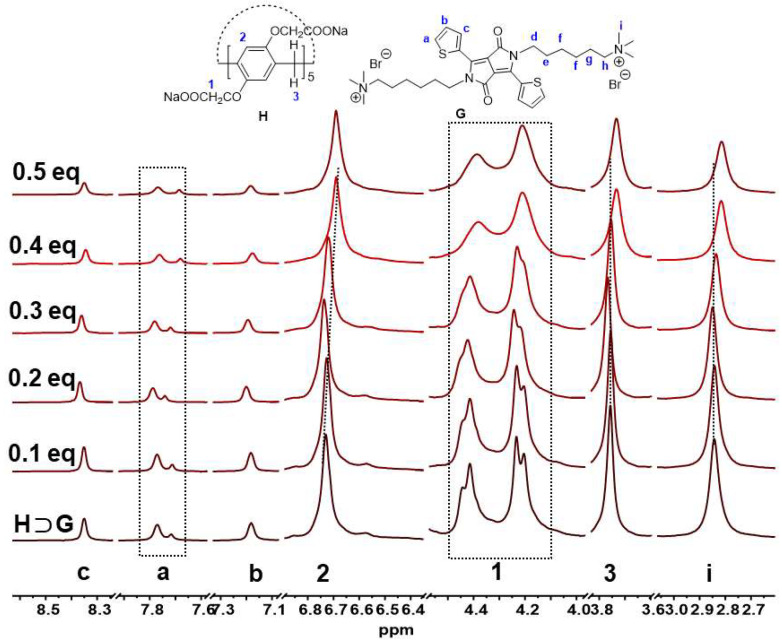
Partial ^1^H NMR titration spectra (500 MHz, D_2_O) of **H⊃G** ([**G**] = 4 mM, [**H**] = 8 mM) with different equivalents of Hg^2+^.

In order to understand the Hg^2+^ detection mechanism by use of **H⊃G** for such a colorimetric and fluorescence phenomenon, the ^1^H NMR spectra of **H⊃G** in D_2_O in the presence of Hg^2+^ were carried out. As shown in Figure 6, proton signals of thienyl (H_a_, H_b_ and H_c_) from **G** showed a slight upfield shift, but H_2_, H_3_ and H_1_ from **H** showed an obvious upfield shift in the presence of Hg^2+^. In addition to this, the splitting peak of H_1_ disappeared, inferring multiple interactions between **H⊃G** and Hg^2+^ were involved. The ^1^H NMR titration spectra suggested that synergistic interactions between Hg^2+^ and host/guest occurred. As we know, the previous work revealed that metal ions could efficiently perturb the microenvironment of host–guest systems [47,48].

At the same time, the control complexation experiment between Hg^2+^ and **H** or **G** alone was investigated through ^1^H NMR, UV-vis and emission spectra. As shown in Figure S9, for free **H**, the methylene protons at both rims (H_1_) were split into two sets of peaks (4.40 and 4.15 ppm) in a 1:1 integration. However, upon the addition of Hg^2+^, the peak H_1_ was merged into a singlet at 4.20 ppm. Meanwhile, the proton signals from the phenyl moieties (H_2_) shifted upfield, and proton signals from the methylene bridge (H_3_) changed from a singlet to a broad peak. The distinct changes indicated an interaction between Hg^2+^ and **H** was present. As shown in Figure S10, ^1^H NMR spectra of **G** showed obvious changes upon the addition of Hg^2+^. The thienyl and N-CH_2_ protons signals of **G** displayed a down-field shift in sharp contrast with pure **G** (Δδ = 0.03, 0.04, 0.03, 0.07 ppm for protons H_c_, H_a_, H_b_, H_d_, respectively), indicating the coordination of Hg^2+^ with S and N atoms of **G** occurred. The ^1^H NMR results were in accordance with a previous study where thienyl DPP could bind to Hg^2+^ with S and N atoms [49]. The presence of Hg^2+^ led to a broad absorption band and an up-shifted baseline of **H** (Figure S11), indicating Hg^2+^ can be bound inside the electron-rich cavity of **H** to form an inclusion complex. On the other hand, Hg^2+^ also induced an obvious change in UV-vis spectra of **G** (Figure S12). As a control, other metal ions failed to take effect on **G** and **H** simultaneously (Figures S13 and S14). As shown in Figure S15, **H⊃G** was stable and had excellent detection performance for Hg^2+^ at pH < 8. Upon increase in pH value, H⊃G was easy to disassemble due to the hydrolysis of G under alkaline conditions. Therefore, under acidic and neutral conditions, the **H⊃G** complex had a good recognition effect on Hg^2+^.

Figure S16 shows the Stern–Volmer plot of **H⊃G** quenched by Hg^2+^. At a low concentration of Hg^2+^, the quenching efficiency of **H⊃G** was more efficient, and I_0_/I vs. the concentration of the quencher gave a linear plot, indicating a static quenching between **H⊃G** and Hg^2+^ was involved.

**Scheme 2:**
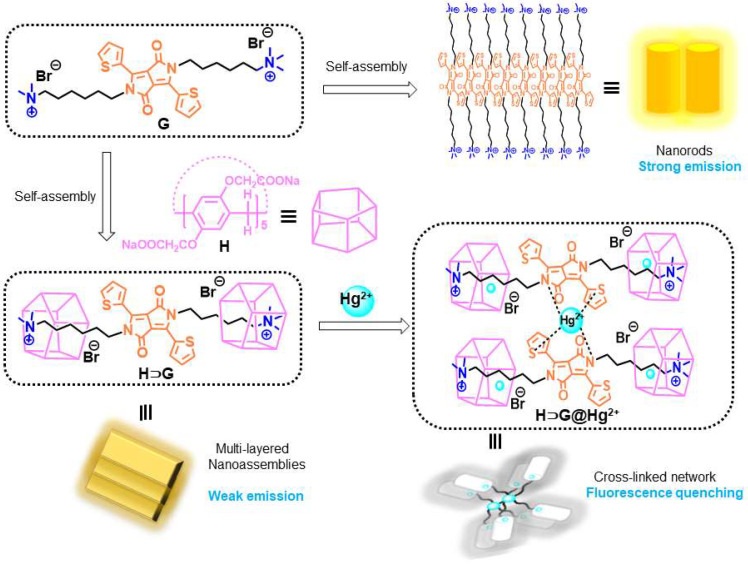
Chemical structures and cartoon representations of carboxylatopillar[5]arene sodium salts (**H**) and diketopyrrolopyrrole-bridged bis(quaternary ammonium) guest (**G**) and the schematic representations of their self-assembly in absence and presence of Hg^2+^ in water.

All these results showed that Hg^2+^ could simultaneously bind **G** and **H** by inclusion and coordination interactions, leading to the high selectivity of Hg^2+^ detection with **H⊃G**. Namely, as a crosslinking agent, Hg^2+^ induced **H⊃G** to perform self-assembly behavior. SEM images confirmed that a denser and crosslinked network was obtained for **H⊃G**@Hg^2+^ (Figure 2e–f), which was different from the multi-layered nanostructure of **H⊃G**. As a result, closer stacking of **H⊃G**@Hg^2+^ gave a more pronounced emission quenching effect. The possible Hg^2+^ detection mechanism was shown in Scheme 2.

### 2.5. Reversibility and Application in the Rapid Removal of Hg^2+^

**Figure 7:**
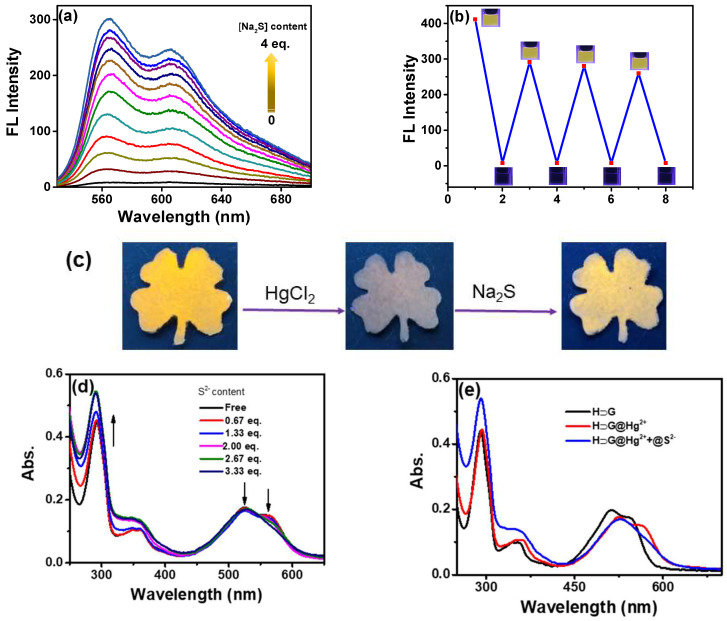
(**a**) Emission spectra of **H⊃G**@Hg^2+^ self-assembly in the presence of increasing amounts of S^2^^−^ (from 0 to 4.0 equivalent in aqueous solution); (**b**) Fluorescent switch of **H⊃G** self-assembly with alternating addition of Hg^2+^and S^2^^−^ (λ_ex_ = 510 nm, λ_em_ = 560 nm, [**H**] = 2 × 10^−5^ M, [**G**] = 10^−5^ M, [Hg^2+^] = 5 × 10^−5^ M, [S^2^^−^]=2 × 10^−4^ M); (**c**) The photos of **H⊃G**-loaded filter paper after exposure of Hg^2+^and subsequent S^2^^−^ under 365 nm irradiation; (**d**) UV-vis spectra of **H⊃G**@Hg^2+^ in the presence of increasing amounts of S^2^^−^; (**e**) UV-vis spectra of **H⊃G**, **H⊃G**@Hg^2+^, **H⊃G**@Hg^2+^@S^2^^−^.

The reversibility of **H⊃G** for Hg^2+^ detection was then evaluated by the addition of Na_2_S. As shown in Figure 7a, the presence of Na_2_S aqueous solution could easily recover the fluorescence of **H⊃G**@Hg^2+^. In addition, with the alternative addition of Na_2_S and Hg^2+^, **H⊃G** showed reversible fluorescence changes (Figure 7b). There was no significant loss of the sensitivity and responsiveness of **H⊃G** after at least four times. When the **H⊃G**-loaded test trip was exposed to Hg^2+^ aqueous solution, an instant change in emission color from yellow to dark was observed. The following S^2^^−^ treatment gave a fluorescence “turn on” response (Figure 7c). SEM images indicated that a denser and crosslinked network of **H⊃G**@Hg^2+^@S^2^^−^ was formed (Figure 2g,h).

The interaction between **H⊃G**@Hg^2+^ and S^2^^−^ was assayed by UV-vis spectral changes. As shown in Figure 7d, the absorption band at 561 nm from **G** gradually disappeared, and the band at 292 nm from **H** blue-shifted to 290 nm upon addition of S^2^^−^, indicating that multiple interactions were involved between **H⊃G**@Hg^2+^ and S^2^^−^. As a control, the **H, G** and **H⊃G** alone further indicated obvious UV-vis spectral responses to S^2^^−^ (Figure S13a–c). From these results, it can be seen that the presence of S^2^^−^ served as both Hg^2+^ adsorption agent and crosslinker to **H⊃G**@Hg^2+^, leading to a denser and more crosslinked network. As shown in Figure 7e, the distinct UV-vis spectra of **H⊃G**, **H⊃G**@Hg^2+^ and **H⊃G**@Hg^2+^@S^2^^−^ were shown.

The performance of **H⊃G** in the applicable removal of Hg^2+^ was also investigated. When the aqueous solution of Hg^2+^ (30 ppm in 10 mL water) was added to the mixture of **H⊃G** (100 μM) in water, the apparent black precipitate was present immediately. The resulting mixture was stirred or ultrasound for another 12 h to guarantee the complete adsorption of the Hg^2+^ ion. Then, residual Hg^2+^ content in the mother liquid after centrifugation was measured by inductively coupled plasma (ICP). The Hg^2+^ removal efficiency was found to be 80.78% and 76.85% under stirring and ultrasound, respectively, which was slightly lower than the reported pillararene supramolecular system in organic solvents [18,19,20,21,22,23,24]. The possible reason can be ascribed that **H⊃G**@Hg^2+^ has higher solubility in water than in organic solvents, leading to higher Hg^2+^ residual in the mother liquid. Some literature revealed that pillararene could enhance the water dispersibility of insoluble or poorly soluble compounds in water [50,51]. However, in our case, the removal of Hg^2+^ was performed in pure water without any organic solvents, which was greener and more economical. Therefore, **H⊃G** maybe have the potential as a versatile absorbent for Hg^2+^.

**Figure 8:**
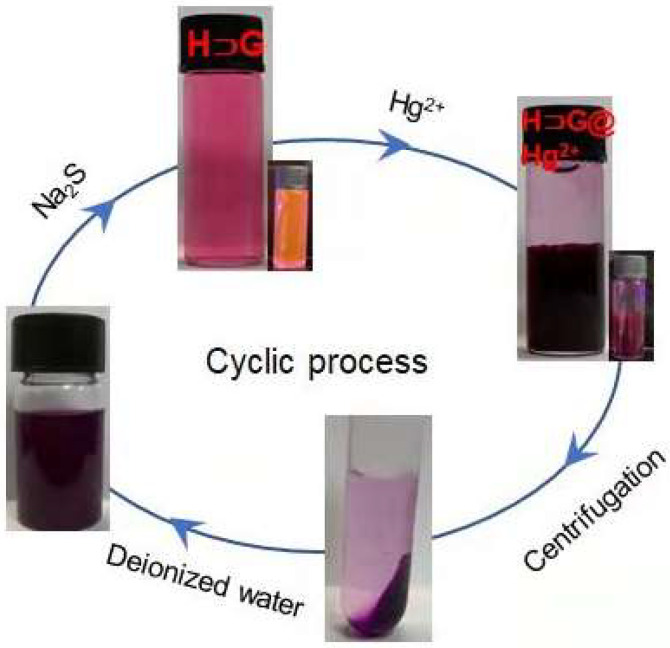
Representative photos during the process of sensing and removal of Hg^2+^ and regeneration of absorbent material (**H⊃G**).

In a regeneration treatment, the separated precipitate **H⊃G**@Hg^2+^ was dissolved in 10 mL of water, followed by the addition of an excessive amount of Na_2_S, and a black HgS solid was produced. The absorbent **H⊃G** was regenerated and recycled after centrifugation (Figure 8).

The practical applicability of **H⊃G** for Hg^2+^ detection was demonstrated by a real sample analysis in tap water and lake water from the South China University of Technology (SCUT). The aliquots of the tap water and lake water were spiked with known amounts of Hg^2+^. As shown in Figure S17, the non-spiked lake water and tap water failed to induce fluorescence change in **H⊃G**. Upon the presence of Hg^2+^, the emission changes at 560 nm of **H⊃G** were quenched, which was plotted on the standard calibration curve in Figure 5b, and the recovery of Hg^2+^ was calculated. As shown in Table 2, up 89.7% recovery was obtained, demonstrating the potential applicability of **H⊃G** for Hg^2+^ detection in the real samples.

## 3. Conclusions

In summary, a fluorescent supramolecular host–guest system (**H⊃G**) of water-soluble carboxylatopillar[5]arene sodium salts (**H**) and a water-soluble DPP derivative (**G**) was conveniently constructed. Morphological transformation of multi-layered nanostructure was achieved during the addition of **H** into **G** aqueous solution, and a fluorescence quenching to some extent was observed. Furthermore, this supramolecular host−guest system (**H⊃G**) could work as a fluorescent probe to selectively and quantitatively detect Hg^2+^ ions through the formation of crosslinked network **H⊃G**@Hg^2+^. With the addition of the Hg^2+^ ion, the fluorescence of **H⊃G** was completely turned off. **H⊃G** showed a reversible response to Hg^2+^ with a detection limit of 7.17 × 10^−7^ M. Our work provided pillararene-based supramolecular fluorescent materials not only for fluorescent detection of Hg^2+^ with high selectivity but also for efficient removal of Hg^2+^.

## Figures and Tables

**Table 1:** The comparison of pillararene-based materials for Hg^2+^ detection.

Pillararene-Based Supramolecular Materials	Detection System, LOD and Sensing Mechanism	Ref.
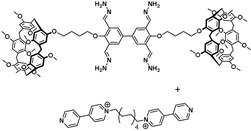	DMSO/H_2_O solution (3:7, *v*/*v*), LOD = 0.1 μM, coordinated with the O atoms of pillar[5]arene and the N atoms of pyridine groups	[43]
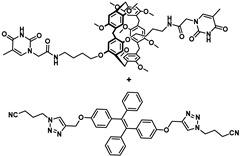	Acetone, LOD = 2.3 μM, unique T−Hg^2+^−T pairings between thymine (T) and Hg^2+^	[18]
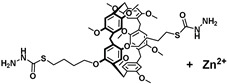	Metal–organic gel, no LOD data, Fluorescence quenching at 470 nm	[44]
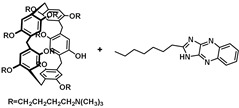	DMSO/H_2_O solution (3:7, *v*/*v*), LOD = 0.5 μM, fluorescence color change from 545 nm to 502 nm	[45]
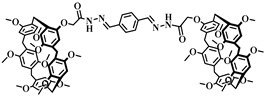	DMSO/H_2_O (1:1, *v*/*v*), LOD = 0.043 μM, Fluorescence quenching at 485 nm, coordination between Hg^2+^ and probe	[46]
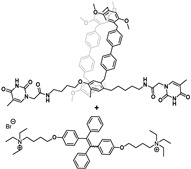	CHCl_3_/acetone/H_2_O (*v*/*v*/*v* = 1:4:495), LOD = 0.03 μM, Unique interaction thymine of **G** and Hg^2+^, Fluorescence turn on at 388 nm.	[21]
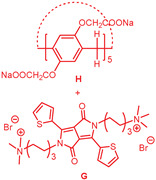	100% water, LOD = 0.7 μM, Fluorescence turn off, interaction with **H** and N, S atoms of **G**, removal and separation of Hg^2+^	This work

**Table 2:** Detection of mercury ion recovery in lake water and tap water.

Sample	Spiked (μM)	Recovered (μM)	Recovery (%)
Lake Water	20.0	17.4	87.0
	26.7	22.3	83.7
	36.7	31.8	86.7
	40.0	34.7	86.8
Tap water	20.0	16.7	83.6
	26.7	22.5	84.5
	30.0	26.9	89.7
	36.7	31.2	85.2
	40.0	35.6	89.0

## Supplementary Materials

The following supporting information [36,41] can be downloaded at: https://www.mdpi.com/article/10.3390/bios12080571/s1, Figure S1: ^1^H NMR spectrum (500 MHz, D_2_O) of **H**; Figure S2: ^13^C NMR spectrum (125 MHz, D_2_O) of compound **H_;_** Figure S3: ^1^H NMR spectrum (500 MHz, D_2_O) of **G**; Figure S4: ^13^C NMR spectrum (125 MHz, D_2_O) of **G**; Figure S5: Normalized UV-vis, emission spectra of **G** in aqueous solution (insert: photographs of aqueous solutions of **G** under daylight and 365 nm light irradiation [**G**] = 10^−5^ M); Figure S6: Binding isotherm of the H⊃G complex fitted with a 1:2 binding model according to fluorescence titration experiment ([**G**] = 10^−5^ M, λ_ex_ = 510 nm); Figure S7: (a) The emission spectra of H⊃G in presence of different metal ions. (b) Plot of fluorescence intnsity changes at 560 nm (ΔI/I_0_) in presence of with the addition of 50.0 equiv. in the presence of 50.0 equiv. of other cations in aqueous solution (Hg^2+^, Cu^2+^, Fe^3+^, Al^3+^, Mn^2+^, Co^2+^, Sn^2+^, Cd^2+^, Ni^2+^); Figure S8: ^1^H NMR titration spectra (500 MHz, D_2_O) of H ([**H**] = 8 mM) with different equivalents of Hg^2+^; Figure S9: Partial ^1^H NMR titration spectra (500 MHz, D_2_O) of **G** ([**G**] = 8 mM) upon addition of Hg^2+^; Figure S10: UV-vis spectra of H upon addition of Hg^2+^ ([**H]** = 10^−5^ M); Figure S11: UV-vis spectra of G upon addition of Hg^2+^([**G**] = 10^−5^ M); Figure S12: UV-vis spectra of (a) G and (b) H upon addition of 5 equiv. of different metal ions ([**G**] = [**H**] = 10^−5^ M); Figure S13: UV-vis spectra of (a) G, (b) H and (c) H⊃G upon addition of various amount of S^2^^−^ ([**G**] = [**H**] = 10^−5^ M, for H⊃G, [**H**] = 10^−5^ M, [**G**] = 2 × 10^−5^ M).
