# Supplementary material for: Green, Efficient Detection and Removal of Hg2+ by Water-Soluble Fluorescent Pillar[5]arene Supramolecular Self-Assembly
Source: Biosensors (Basel). 2022 Jul 27;12(8):571. doi: 10.3390/bios12080571 (PMC9405992; doi:10.3390/bios12080571)
Supplement: Supplementary file 1 [file biosensors-12-00571-s001.zip › biosensors-1796930-supplementary.pdf]

Article

Green, Efficient Detection and Removal of Hg^{2+} by Water-soluble Fluorescent Pillar[5]arene Supramolecular Self-assembly

Xiaomei Jiang [1], Lingyun Wang [1,*], Xueguang Ran [2], Hao Tang [1] and Derong Cao [1]

1 Key Laboratory of Functional Molecular Engineering of Guangdong Province, School of Chemistry and Chemical Engineering, South China University of Technology, 381 Wushan Road, Guangzhou 510641, China; 201910104286@mail.scut.edu.cn (X.J.); haotang@scut.edu.cn (H.T.); drcao@scut.edu.cn (D.C.)

2 Institute of Animal Science, Guangdong Academy of Agricultural Sciences, Ministry of Agriculture Key Laboratory of Animal Nutrition and Feed Science in South China, State Key Laboratory of Livestock and Poultry Breeding, Guangzhou 510641, China; rxg59@aliyun.com

* Correspondence: lingyun@scut.edu.cn

Experimental Characterizations

The UV-Vis absorption spectra were recorded using a Helios Alpha UV-Vis scanning spectrophotometer with a 1 cm quartz cell. Fluorescence spectra were quantitatively measured by FluoroMax -4 spectrofluorometer with a xenon lamp and 0.5 cm quartz cells. High-resolution mass spectra were carried on LCQ Fleet LC-MS System (Thermo Fisher Scientific). Mass spectra were recorded on Waters ACQUITY TQD liquid chromatograph–mass spectrometer using APCI ionization. ^{1}H NMR and ^{13}C NMR spectra were carried on a Bruker spectrometer. Compounds **P5A2** and **DPP-Br** were prepared according to the previous method, respectively [S1, S2].

Synthesis of host molecule H and guest molecule G

Synthesis of compound **H**

Compound **P5A2** (735 mg, 0.5 mmol) was dispersed in ethanol (50 mL). Then, NaOH solution (400 mg NaOH in 0.5 mL of deionized H_2O) was added to the mixture. The mixture was stirred at 80 °C under an N_2 atmosphere for 8 h, during which a white precipitate was formed. After cooling to room temperature, the precipitate was filtered and washed with a mixed solution of ethanol and water (100/1, v/v) to afford pure compound **H** as a white solid (85% yield, 600 mg). ^{1}H NMR (D_2O, 500 MHz, δ, ppm): 6.70 (s, 10 H), 4.46-4.43 (d, *J* = 16 Hz, 10 H), 4.17-4.14 (d, *J* = 16 Hz, 10 H), 3.74 (s, 10 H). ^{13}C NMR (125 MHz, D_2O) *δ* 177.50, 149.17, 128.48, 114.35, 67.62, 28.96.

Synthesis of compound **G**

Compound **DPP-Br** (220 mg, 0.351 mmol) and trimethylamine (83 mg, 1.404 mmol) were dissolved in chloroform (10 mL). The mixture was stirred at 65 °C under an N_2 atmosphere for 10 h, during which a red precipitate was formed. After cooling, the precipitate was filtered and washed with chloroform to result in a red solid **G** (98% yield, 250 mg). ^{1}H NMR (500 MHz, D_2O) δ 8.33-8.32 (d, J = 3.8 Hz, 2 H), 7.78-7.77 (d, *J* = 4.9 Hz, 2 H), 7.19-7.17 (t, *J* = 4.4 Hz, 2 H), 3.55-3.51 (t, *J* = 7.9 Hz, 4 H), 3.25-3.21 (m, 4 H), 3.08 (s, 18 H). 1.72-1.67 (m, 8 H), 1.43-1.40 (m, 4 H), 1.26 (s, 8 H). ^{13}C NMR (125 MHz, D_2O) δ 160.97, 140.21, 135.18, 133.06, 128.69, 106.35, 66.48, 52.84, 41.85, 28.51, 25.45, 24.92, 22.27.

The host-guest complexation by ^{1}H NMR titration experiment

The **G** solution in D_2O (8.3 mM) was firstly prepared. Then, different concentration of **H** in D_2O (up to 33.2 mM) was added. After 5 min, the ^{1}H NMR spectrum was recorded.

Experimental procedure of H⊃G for Hg^{2+} detection

The **H⊃G** (10 μM) in water was mixed with different concentrations of Hg^{2+} aqueous solution at 25 °C for 1 min. Individual samples for spectral data were recorded. The resulting individual solution was shaken well for absorption and fluorescence spectral analysis.

The emission color change of H⊃G loaded filter paper in presence of Hg^{2+} and following S^{2-}

A volume of 200 μL of water stock solution of the **H⊃G** (1 mM) was drop-casted onto the Whatman filter paper, followed by evaporation to dry. The **H⊃G** loaded filter paper was exposed to Hg^{2+} aqueous solutions for 1 min, and following treatment with S^{2-}. Then the emission color change under 365 nm irradiation was measured with a camera.

Measurement of detection limit

The detection limit was gained from the fluorescence titration data. On the basis of the results of the emission titrating experiment, a good linear relationship between the emission at 560 nm of **H⊃G** and Hg^{2+} concentrations ranging from 10 to 40 μM was obtained. A limit of detection (LOD) was calculated by means of Eq. (1):

Detection limit = $(3\times\sigma)/k$ (1)

Where σ is the standard deviation of blank measurements, k is the slope between emission at 560 nm versus Hg^{2+} concentration.

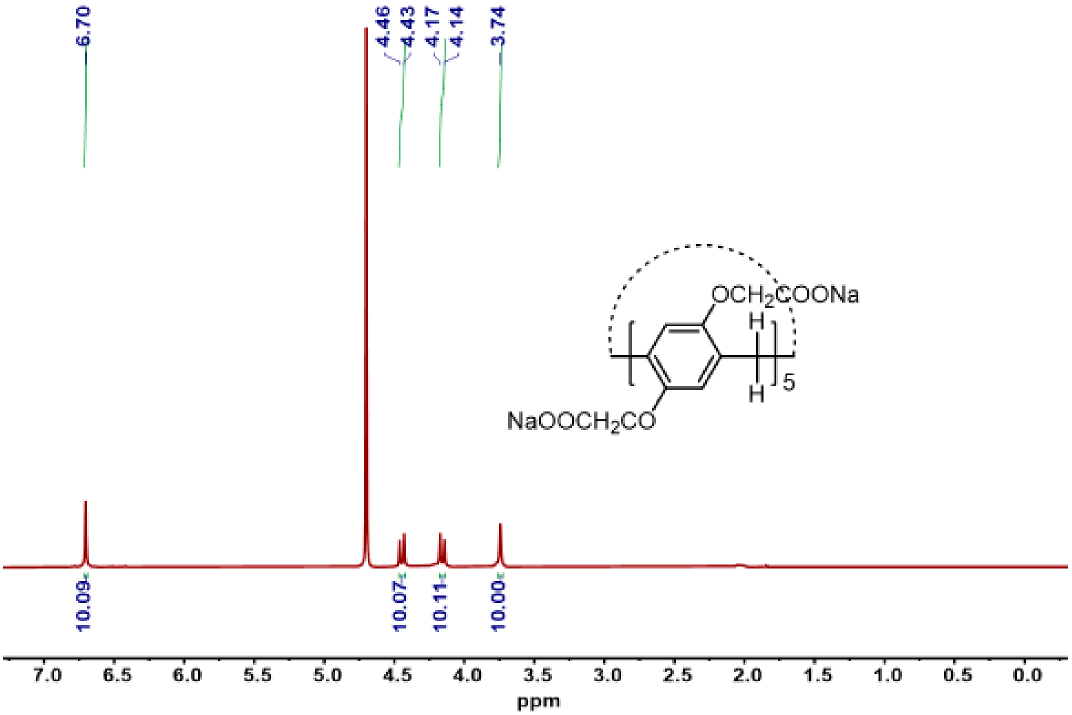

Figure S1. ^1H NMR spectrum (500 MHz, D_2O) of **H**.

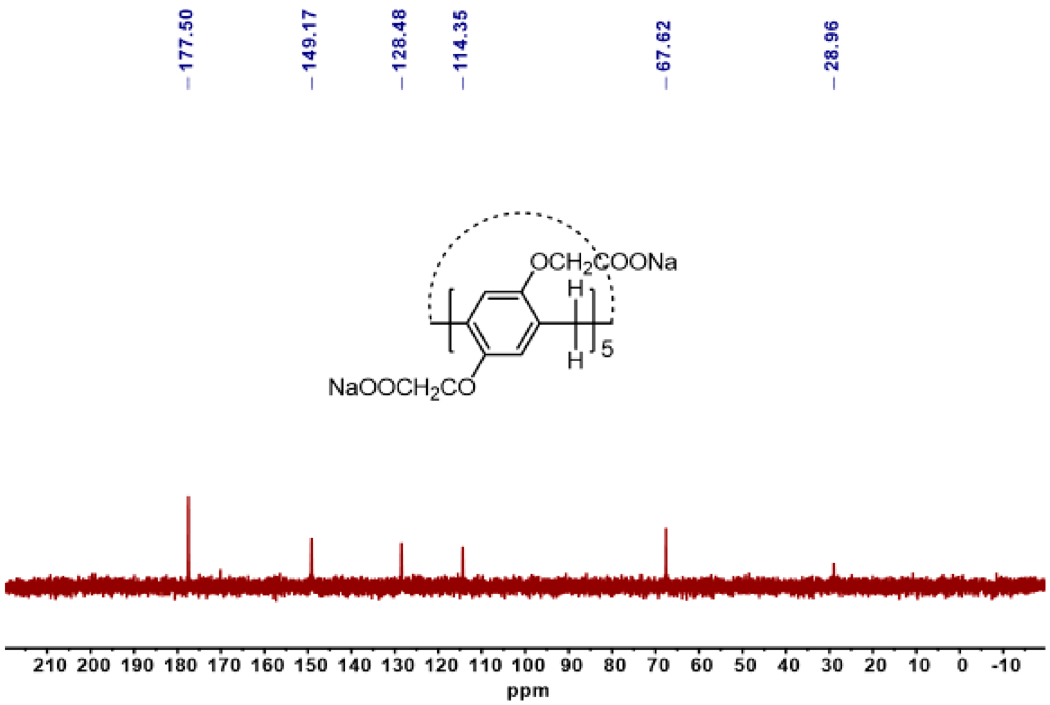

Figure S2. ^{13}C NMR spectrum (125 MHz, D_2O) of compound **H**.

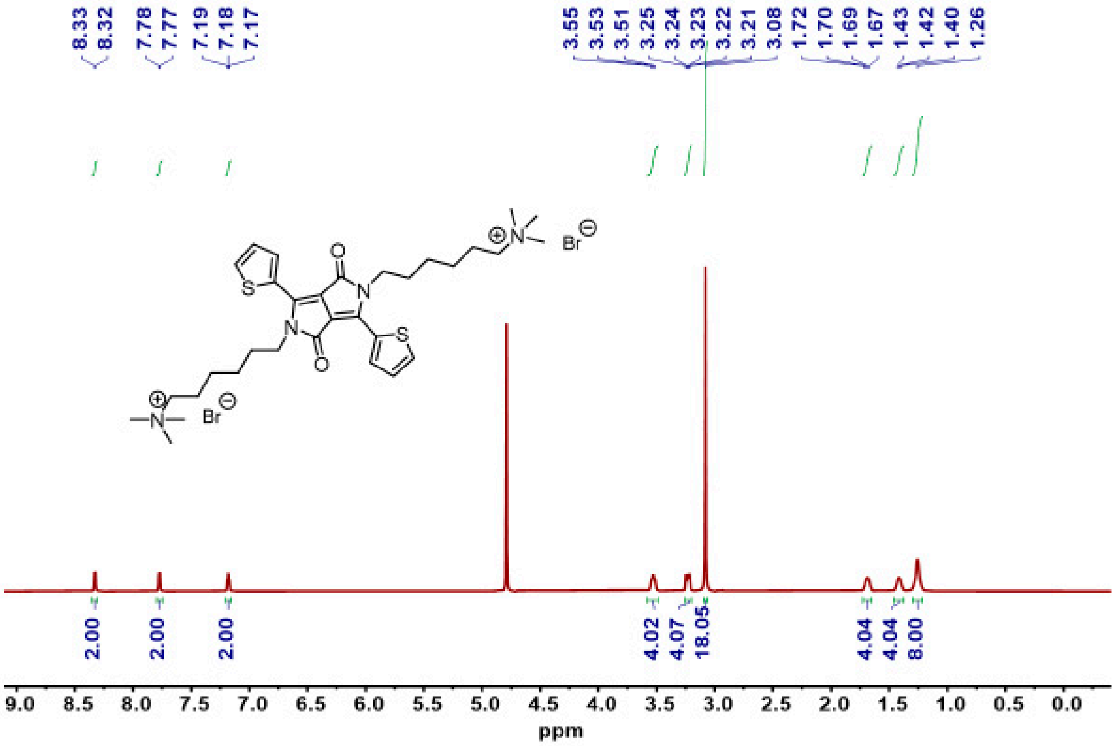

Figure S3. ^{1}H NMR spectrum (500 MHz, D_2O) of **G**.

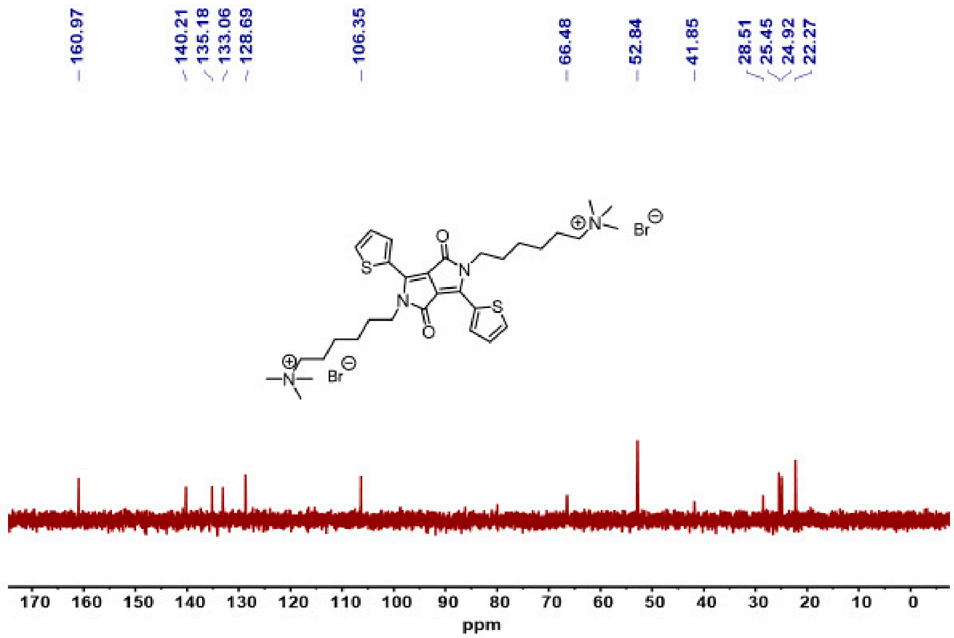

Figure S4. ^{13}C NMR spectrum (125 MHz, D_2O) of **G**.

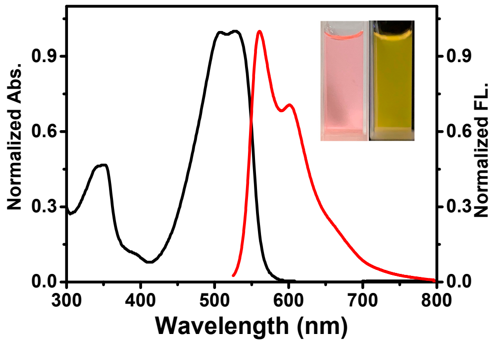

Figure S5. Normalized UV-vis emission spectra of **G** in aqueous solution (insert: photographs of aqueous solutions of **G** under daylight and 365 nm light irradiation [**G**]=10^{-5} M).

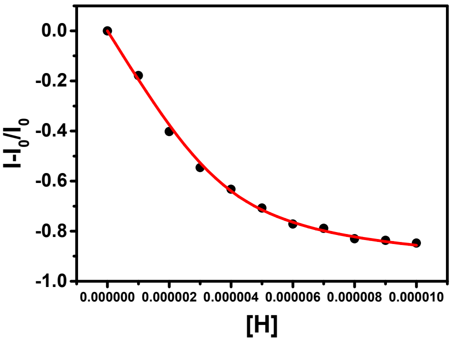

Figure S6. Binding isotherm of the **H⊃G** complex fitted with a 1:2 binding model according to fluorescence titration experiment ([**G**]=10^{-5} M, λ_{ex} = 510 nm).

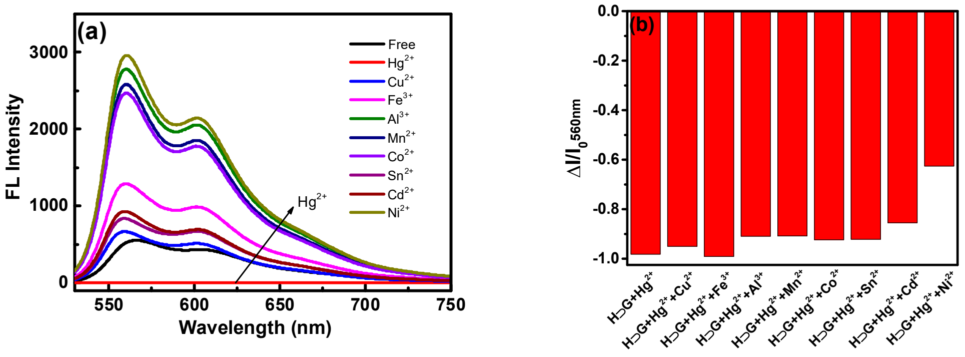

Figure S7. (a) The emission spectra of **H⊃G** in presence of different metal ions. (b) Plot of fluorescence intensity changes at 560 nm ($\Delta I/I_0$) in presence of with the addition of 50.0 equiv. in the presence of 50.0 equiv. of other cations in aqueous solution (Hg^{2+}, Cu^{2+}, Fe^{3+}, Al^{3+}, Mn^{2+}, Co^{2+}, Sn^{2+}, Cd^{2+}, Ni^{2+}).

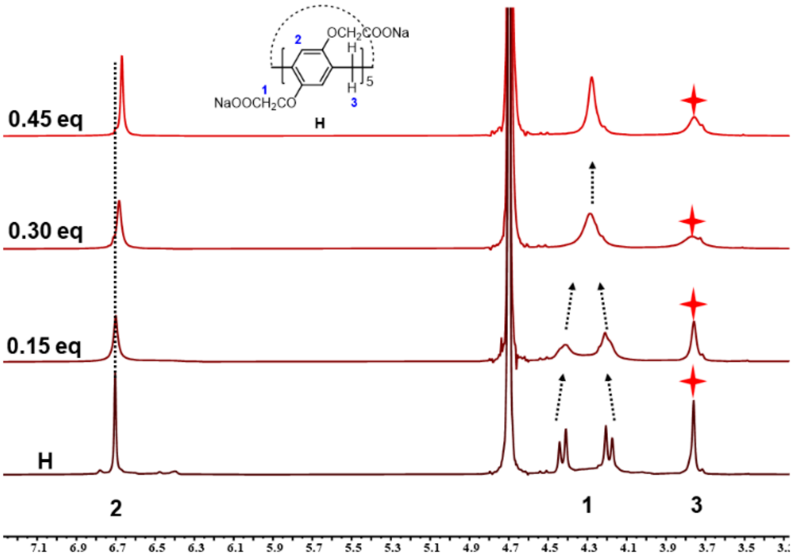

Figure S8. ^1H NMR titration spectra (500 MHz, D_2O) of **H** ([**H**] = 8 mM) with different equivalents of Hg^{2+}.

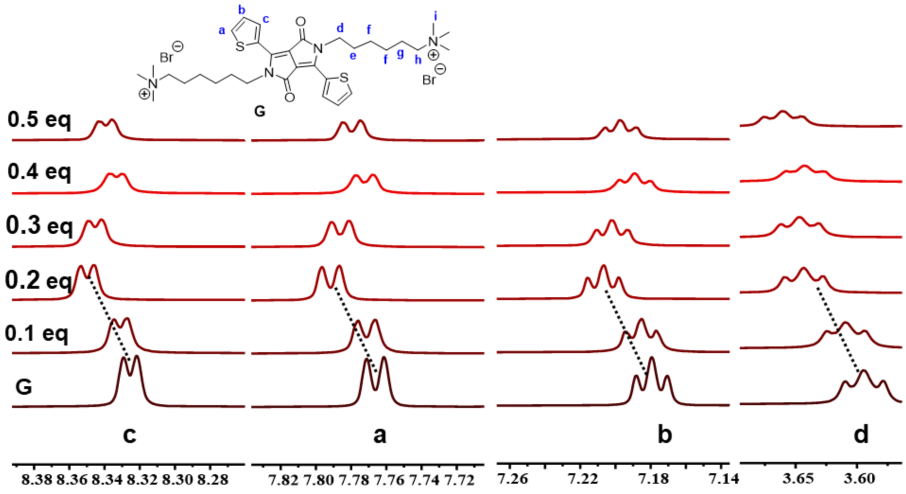

Figure S9. Partial ^1H NMR titration spectra (500 MHz, D_2O) of **G** ([**G**] = 8 mM) upon addition of Hg^{2+}.

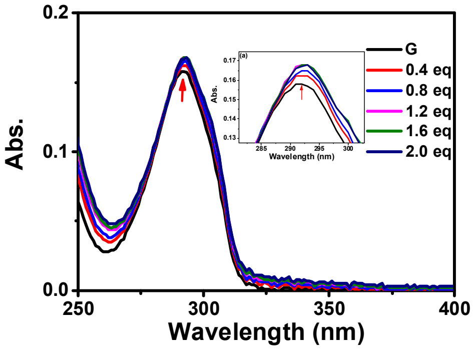

Figure S10. UV-vis spectra of **H** upon addition of Hg^{2+} ([**H**]=10^{-5} M).

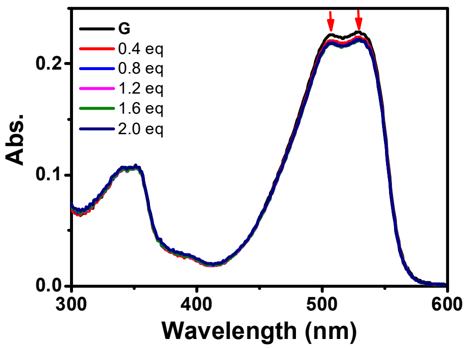

Figure S11. UV-vis spectra of **G** upon addition of Hg^{2+}([**G**]=10^{-5} M).

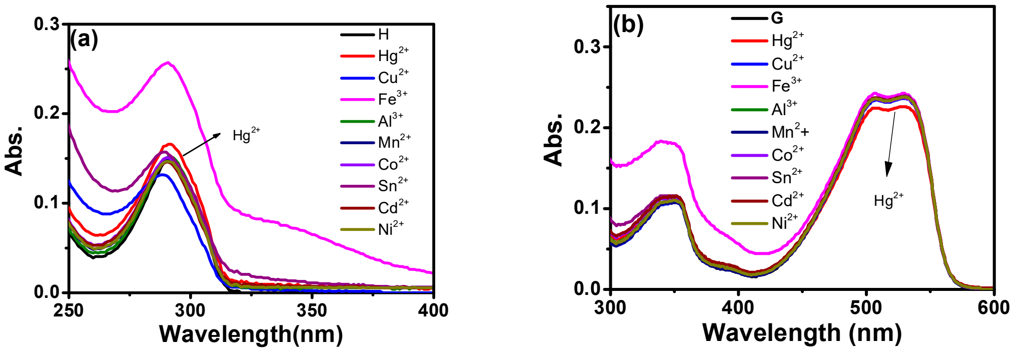

Figure S12. UV-vis spectra of (a) **G** and (b) **H** upon addition of 5 equiv. of different metal ions ([**G**] = [**H**] = 10^{-5} M).

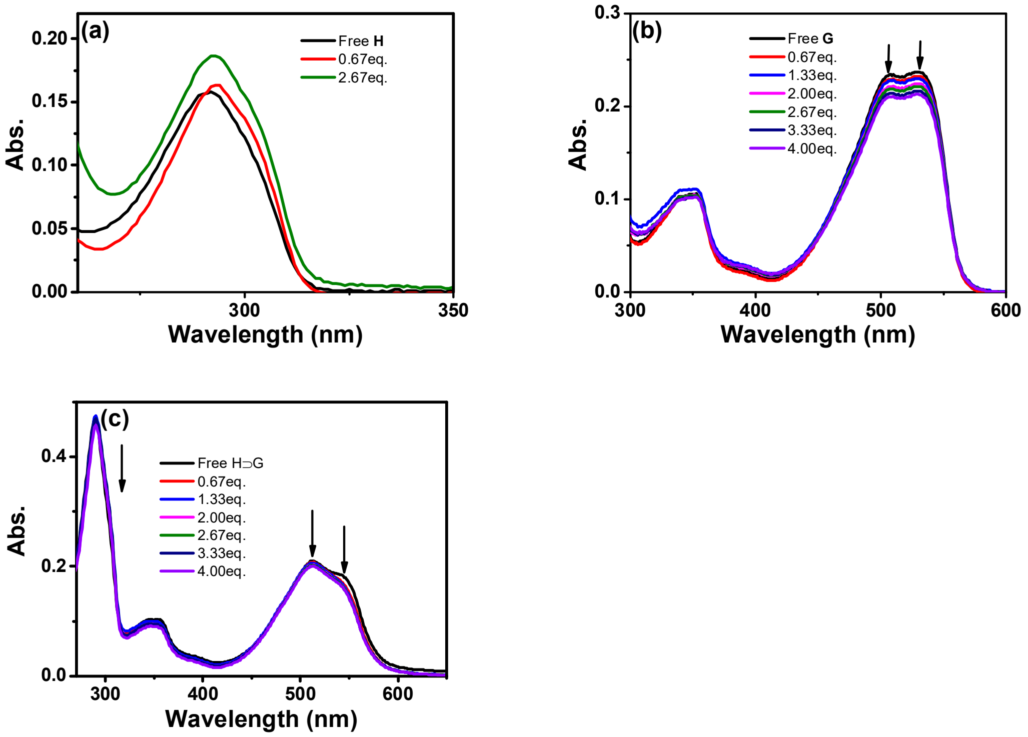

Figure S13. UV-vis spectra of (a) **G**, (b) **H** and (c) **H⊃G** upon addition of various amount of S^{2-} ([**G**] = [**H**] = 10^{-5} M, for **H⊃G**, [**H**] = 10^{-5} M, [**G**] = 2×10^{-5} M)
